# Supplementary material for: Management of locally advanced non-small cell lung cancer in the modern era: A national Italian survey on diagnosis, treatment and multidisciplinary approach
Source: PLoS One. 2019 Nov 13;14(11):e0224027. doi: 10.1371/journal.pone.0224027 (PMC6853329; doi:10.1371/journal.pone.0224027)
Supplement: S1 Appendix — (DOCX) [file pone.0224027.s001.docx]

**S1 Appendix – Questionnaire**

1. **What is your specialty?**
2. Radiation Oncology
3. Medical Oncology
4. Pneumology
5. Thoracic Surgery
6. Other
7. **How many years have you been exercising??**
8. 0-5 years
9. 5-10 years
10. 10-15 years
11. >15 years
12. **In which hospital structure do you work?**
13. General hospital
14. Academic
15. IRCCS – Cancer Care Center
16. Private
17. Other
18. **What percentage is dedicated to pulmonary oncology pathology?**
19. 90-100%
20. 70-90%
21. 50-70%
22. < 50%
23. **In which geographic area you practice your profession**:
24. North
25. Central Italy
26. South
27. Islands
28. **In your Center who is in charge of the patient in the diagnostic and staging phase?**
29. Pneumologist
30. Radiation Oncologist
31. Medical Oncologist
32. Thoracic Surgeon
33. Other
34. **Is there a multidisciplinary group in your Center to discuss and take care of patients with lung cancer?**
35. Yes, with weekly meetings
36. Yes, with meetings every two weeks
37. Yes, but not regularly
38. None
39. **How many patients with locally advanced NSCLC (IIIA-IIIB) as the first diagnosis have been assigned to your department in the last year??**
40. >30 patients
41. 20-30 patients
42. 10-20 patients
43. < 10 patients
44. **In a patient with newly LA-NSCLC diagnosis with lymph nodal mediastinal PET positivity, which method do you use to complete the staging?**
45. None
46. TBNA
47. TBNA/EBUS
48. Mediastinoscopy
49. **In a patient with newly LA-NSCLC diagnosis with lymph nodal mediastinal PET negativity, which method you use for staging?**

a) None

b) TBNA

c) TBNA/EBUS

d) Mediastinoscopy

1. **Which biological characterization do you consider mandatory before planning a radical treatment of LA-NSCLC?**
2. Positive cytology
3. histological differential diagnosis between adenocarcinoma and squamous cell carcinoma
4. Bio-molecular characterization (EGFR, ALK, ROS1)
5. c + PDL1
6. **Which therapeutic approach would you recommend in a patient with lung adenocarcinoma in clinical stage T1bcN2 (single station involvement), IIIA, fit for surgery?**
7. Upfront surgery
8. Neoadjuvant chemotherapy followed by surgery
9. Neoadjuvant chemo-radiation followed by surgery
10. chemo-radiation at radical intent
11. **Which therapeutic approach would you recommend in a patient with lung adenocarcinoma in clinical stage cT2cN2 (multiple positive lymph node stations), IIIA, fit for surgery?**
12. Upfront surgery
13. Neoadjuvant chemotherapy followed by surgery
14. Neoadjuvant chemo-radiation followed by surgery
15. chemo-radiation at radical intent
16. **Which therapeutic approach would you recommend in a patient with lung adenocarcinoma inoperable at diagnosis in partial response/stability (ycN2) after neoadjuvant chemotherapy?**
17. upfront surgery only if lobectomy is feasible
18. upfront surgery in any case
19. chemo-radiation (if not performed in neoadjuvant setting)
20. radiotherapy (if not performed in neoadjuvant setting)
21. chemotherapy
22. **Which therapeutic approach would you recommend in a patient candidate for chemo-radiation treatment with stage IIIA-B NSCLC?**
23. Concurrent chemo-radiation
24. Concurrent chemo-radiation followed by chemotherapy
25. Sequential chemo-radiation
26. Chemotherapy alone

**16) Why do not you recommend the concomitant approach if the patient is fit for this treatment?**

1. internal protocol
2. logistic issues
3. acute toxicity risk
4. lack of a multidisciplinary approach to diagnosis
