# Supplementary material for: Management of locally advanced non-small cell lung cancer in the modern era: A national Italian survey on diagnosis, treatment and multidisciplinary approach
Source: PLoS One. 2019 Nov 13;14(11):e0224027. doi: 10.1371/journal.pone.0224027 (PMC6853329; doi:10.1371/journal.pone.0224027)
Supplement: S2 Appendix — (DOCX) [file pone.0224027.s002.docx]

**S2 Appendix : Correct answer accepted by NSCLC Experts**

| **Questions** | **Right answer** |
| --- | --- |
| ***In a patient with newly LA-NSCLC diagnosis with lymph nodal mediastinal PET positivity, which tool do you use to complete the staging?*** | TBNA/EBUS |
| ***In a patient with newly LA-NSCLC diagnosis with lymph nodal mediastinal PET negativity, which method do you use to complete the staging?*** | TBNA/EBUS |
| ***Which biological characterization do you consider mandatory to plan radical treatment of LA-NSCLC?*** | Histological differential diagnosis between adenocarcinoma and squamous cell carcinoma * |
| ***Which therapeutic approach would you recommend in a patient with lung adenocarcinoma in clinical stage T1bcN2 (monostation involvement), Stage IIIA, fit for surgery?*** | Neoadjuvant chemotherapy followed by surgery |
| ***Which therapeutic approach would you recommend in a patient with lung adenocarcinoma in clinical stage cT2cN2 (pluristation involvement), stage IIIA, fit for surgery?*** | Chemo-radiation therapy |
| ***Which therapeutic approach would you recommend in a patient with unresectable lung adenocarcinoma at time of diagnosis in partial response/stability (ycN2) after neoadjuvant chemotherapy?*** | Chemo-radiation therapy |
| ***Which therapeutic approach would you recommend in a patient candidate for chemo-radiation treatment with stage IIIA-B lung cancer?*** | Concurrent approach |

* Before PACIFIC trial results
