# Supplementary material for: Management of locally advanced non-small cell lung cancer in the modern era: A national Italian survey on diagnosis, treatment and multidisciplinary approach
Source: PLoS One. 2019 Nov 13;14(11):e0224027. doi: 10.1371/journal.pone.0224027 (PMC6853329; doi:10.1371/journal.pone.0224027)
Supplement: S3 Appendix — (DOCX) [file pone.0224027.s003.docx]

**Appendix S3**

**Statistical analysis for diagnostic management comparing subgroups and entire population**

| Questions |  | In a patient with newly LA-NSCLC diagnosis with lymph nodal mediastinal PET *positivity*, which method you use for stadiative completion? | In patient with newly LA-NSCLC diagnosis with lymph nodal mediastinal PET *negativity*, which method you use for stadiative completion? | Which biological characterization do you consider mandatory for the radical treatment of LA-NSCLC? |
| --- | --- | --- | --- | --- |
| **Subgroups** | **N** | **Number of 1-2-3-4 answers (P Value)** | | |
| **Specialization:**  Radiation Oncology  Medical Oncology  Pneumology  Thoracic Surgery | 165  81  86  64 | 60-8-89-8 (p=0.00)  19-5-53-4 (p=0.828)  9-18-55-4 (p=0.00)  4-4-54-2 (p=0.01) | 37-11-100-17 (p=0.15)  22-6-46-7 (p=0.77)  13-16-51-6 (p=0.007)  22-7-32-3 (p=0.145) | 25-78-43-19 (p=0.049)  22-44-8-7 (p=0.001)  15-22-28-21 (p=0.00)  16-26-10-12 (p=0.302) |
| **Level of experience:**  0- 5 years  5-10 years  10-15 years  > 15 years | 117  48  78  156 | 26-7-77-7 (p=0.476)  15-2-28-3 (p=0.336)  14-4-58-2 (p=0.150)  38-22-90-6 (p=0.018) | 22-8-76-11 (p=0.370)  11-4-27-6 (p=0.147)  19-5-49-5 (p=0.709)  44-23-78-11 (p=0.547) | 25-53-22-17 (p=0.165)  7-23-11-7 (p=0.625)  21-31-16-10 (p=0.787)  25-63-42-26 (p=0.331) |
| **Dedicated working time:**  90-100%  70-90%  50-70%  <50% | 49  84  106  160 | 10-5-32-2 (p=0.943)  12-8-61-3 (p=0.144)  18-8-76-4 (p=0.215)  53-14-84-9 (p=0.001) | 15-5-25-4 (p=0.014)  19-9-50-6 (p=0.946)  30-8-62-6 (p=0.366)  32-18-93-17 (p=0.258) | 16-26-2-5 (p=0.233)  17-40-10-17 (p=0.04)  20-47-19-20 (p=0.384)  25-57-60-18 (p=0.00) |
| **Frequency of MTD:**  weekly  bi-weekly  not regularly  none | 288  34  38  39 | 56-25-195-12 (p=0.019)  6-3-23-2 (p=0.856)  13-4-19-2 (p=0.317)  18-3-16-2 (p=0.004) | 67-28-174-19 (p=0.154)  10-2-16-6 (p=0.124)  8-6-22-2 (p=0.570)  11-4-18-6 (p=0.267) | 60-124-51-53 (p=0.00)  6-17-10-1 (p=0.181)  4-18-14-2 (p=0.041)  8-11-16-4 (p=0.028) |
| **N of LA-NSLC pts in last year**  > 30  20-30  10-20  < 10 | 137  105  112  45 | 27-14-90-6 (p=0.607)  20-6-76-3 (p=0.151)  35-10-60-7 (p=0.054)  11-5-27-2 (p=0.932) | 36-15-82-4 (p=0.046)  22-8-65-10 (p=0.549)  24-13-61-14 (p=0.214)  14-4-22-5 (p=0.516) | 24-55-28-30 (p=0.051)  16-51-25-13 (p=0.351)  26-48-23-15 (p=0.636)  12-16-15-2 (p=0.04) |
