# Supplementary material for: Management of locally advanced non-small cell lung cancer in the modern era: A national Italian survey on diagnosis, treatment and multidisciplinary approach
Source: PLoS One. 2019 Nov 13;14(11):e0224027. doi: 10.1371/journal.pone.0224027 (PMC6853329; doi:10.1371/journal.pone.0224027)
Supplement: S4 Appendix — (DOCX) [file pone.0224027.s004.docx]

**Appendix S4**

**Statistical analysis for therapeutic management comparing subgroups and entire population**

| Questions |  | Which therapeutic approach would you recommend in a patient with NSCLC in stage T1bN2 (*monostation involvement*), IIIA, fit for surgery? | Which therapeutic approach would you recommend in patient with NSCLC in clinical stage T1bN2 (*no bulky pluristation involvement*), IIIA, fit for surgery? | | Which therapeutic approach would you recommend in patient with NSCLC inoperable at diagnosis in partial response/stability (ycN2) after neoadjuvant cht? | Which therapeutic approach would you recommend in a patient candidate for chemo-radiation treatment with stage IIIA-B lung cancer? |
| --- | --- | --- | --- | --- | --- | --- |
| **Subgroups** | **N** | **Number of 1-2-3-4-5 answers (P Value)** | | | |  |
| **Specialization:**  Radiation Oncology  Medical Oncology  Pneumology  Thoracic Surgery | 165  81  86  64 | 39-54-30-18-24 (p=0.00)  18-45-4-4-10 (p=0.036)  6-45-17-1-17 (p=0.00)  28-26-1-0-9 p(0.00) | | 4-36-27-73-25 (p=0.00)  1-25-8-29-18 (p=0.421)  2-21-22-19-22 (p=0.046)  0-26-7-5-26 (p=0.00) | 24-2-95-40-4 (p=0.00)  14-2-40-23-2 (p=0.057)  21-0-35-11-19 (p=0.00)  29-6-23-2-4 (p=0.00) | 107-44-14-0 (p=0.01)  49-21-11-0 (p=0.241)  38-34-14-0 (p=0.11)  21-33-10-0 (p=0.001) |
| **Level of experience:**  0- 5 years  5-10 years  10-15 years  > 15 years | 117  48  78  156 | 33-38-17-7-22 (p=0.056)  9-21-7-3-8 (p=0.109)  20-32-13-4-9 (p=0.969)  29-79-17-9-22 (p=0.725) | | 2-34-19-34-28 (p=0.308)  0-10-8-17-13 (p=0.962)  3-20-14-24-17 (p=0.675)  2-44-24-51-35 (p=0.597) | 23-3-62-20-9 (p=0.381)  16-0-17-10-5 (p=0.853)  12-4-39-18-5 (p=0.124)  37-3-77-28-11 )p=0.229) | 55-51-11-0 (p=0.05)  29-14-5-0 (p=0.027)  39-26-13-0 (p=0.626)  92-44-20-0 (p=0.407) |
| **Dedicated working time:**  90-100%  70-90%  50-70%  <50% | 49  84  106  160 | 9-27-4-1-8 (p=0.115)  30-44-3-3-4 (p=0.00)  20-45-15-5-21 (p=0.520)  32-54-32-14-28 (p=0.001) | | 1-11-7-14-16 (p=0.944)  0-34-15-16-19 (p=0.006)  2-31-16-37-20 (p=0.714)  4-32-27-59-38 (p=0.084) | 16-4-19-9-1 (p=0.922)  23-5-36-12-8 (p=0.060)  18-0-57-23-8 (p=0.162)  31-1-83-32-13 (p=0.249) | 26-17-6-0 (p=0.158)  39-32-13-0 (p=0.279)  62-32-12-0 (0.537)  88-54-18-0 (p=0.864) |
| **Frequency of MTD:**  weekly  bi-weekly  not regularly  none | 288  34  38  39 | 68-125-32-18-45 (p=0.245)  5-17-8-0-4 (p=0.151)  10-14-4-5-5 (p=0.293)  8-14-10-0-7 (p=0.094) | | 6-82-39-92-69 (p=0.169)  0-9-11-5-9 (p=0.040)  0-9-5-16-8 (p=0.603)  1-8-10-13-7 (p=0.442) | 69-9-128-60-22 (p=0.057)  7-0-21-4-2 (p=0.493)  5-0-27-4-2 (p=0.070)  7-1-19-8-4 (p=0.937) | 161-96-31-0 (p=0.247)  14-14-6-0 (p=0.276)  16-16-6-0 (p=0.309)  24-9-6-0 (p=0.319) |
| **N of LA-NSLC pts in last year**  > 30  20-30  10-20  < 10 | 137  105  112  45 | 27-67-16-10-17 (p=0.255)  27-48-15-5-10 (p=0.372)  26-40-17-6-23 (p=0.297)  11-15-6-2-11 )p=0.403) | | 3-36-30-39-29 (p=0.253)  2-28-14-35-26 (p=0.901)  2-32-10-40-28 (p=0.175)  0-12-11-12-10 (p=0.497) | 31-5-62-26-13 (p=0.6)  25-3-49-21-7 (p=0.961)  23-1-61-22-5 (p= 0.315)  9-1-23-7-5 (p=0.851) | 69-48-20-0 (p=0.477)  55-35-15-0 (p=0.765)  63-40-9-0 (p=0.271)  28-12-5-0 (p=0.477) |
