# Supplementary material for: Management of locally advanced non-small cell lung cancer in the modern era: A national Italian survey on diagnosis, treatment and multidisciplinary approach
Source: PLoS One. 2019 Nov 13;14(11):e0224027. doi: 10.1371/journal.pone.0224027 (PMC6853329; doi:10.1371/journal.pone.0224027)
Supplement: S5 Appendix — (DOCX) [file pone.0224027.s005.docx]

**Appendix S5**

**Table. Statistical analysis for diagnostic management comparing subgroups and correct answers**

| Questions |  | In a patient with newly LA-NSCLC diagnosis with lymph nodal mediastinal PET positivity, which method you use for stadiative completion? | In a patient with newly LA-NSCLC diagnosis with lymph nodal mediastinal PET negativity, which method you use for stadiative completion? | Which biological characterization do you consider mandatory for the radical treatment of LA-NSCLC? |
| --- | --- | --- | --- | --- |
| **Subgroups** | **N** | **Right answers % (P Value)** | | |
| Specialization:  Radiation Oncology  Medical Oncology  Pneumology  Thoracic Surgery | 165  81  86  64 | 54 (p=0.001)  65 (p=0.672)  64 (p=0.96)  84 (p =0.00) | 61 (p=0.315)  57 (p=0.862)  59 (p=0.725)  50 (p=0.177) | 47 (p=0.113)  54 (p=0.017)  26 (p=0.00)  41 (p=0.726) |
| Level of experience:  0- 5 years  5-10 years  10-15 years  > 15 years | 117  48  78  156 | 66 (p=0.521)  58 (p=0.436)  74 (p=0.025)  58 (p=0.058) | 65 (p=0.057)  56 (p=0.835)  63 (p=0.302)  50 (p=0.013) | 45 (p=0.484)  48 (p=0.428)  40 (p=0.569)  40 (p=0.472) |
| Dedicated working time:  90-100%  70-90%  50-70%  <50% | 49  84  106  160 | 65 (p=0.768)  73 (p=0.049)  72 (p=0.039)  48 (p=0.00) | 51 (p=0.316)  60 (p=0.695)  58 (p=0.837)  58 (p= 0.874) | 53 (p=0.114)  48 (p=0.296)  44 (p=0.674)  36 (p=0.021) |
| Frequency of MTD:  weekly  bi-weekly  not regularly  none | 288  34  38  39 | 68 (p=0.004)  68 (p=0.592)  50 (p=0.071)  41 (p=0.002) | 60 (p=0.071)  47 (p=0.192)  58 (p=0.974)  46 (p=0.126) | 43 (p=0.770)  50 (p=0.362)  47 (p=0.533)  28 (p=0.056) |
| N of LA-NSLC pts in last year  > 30  20-30  10-20  < 10 | 137  105  112  45 | 66 (p=0.493)  72 (p=0.026)  54 (p=0.011)  60 (p=0.614) | 60 (p=0.518)  62 (p=0.303)  54 (p=0.422)  49 (p=0.207) | 40 (p=0.472)  49 (p=0.150)  43 (p=0.950)  36 (p=0.310) |
