# Supplementary material for: Management of locally advanced non-small cell lung cancer in the modern era: A national Italian survey on diagnosis, treatment and multidisciplinary approach
Source: PLoS One. 2019 Nov 13;14(11):e0224027. doi: 10.1371/journal.pone.0224027 (PMC6853329; doi:10.1371/journal.pone.0224027)
Supplement: S6 Appendix — (DOCX) [file pone.0224027.s006.docx]

**Appendix S6**

**Table : Statistical analysis for therapeutic management comparing subgroups and correct answers**

| Questions |  | Which therapeutic approach would you recommend in a patient with lung adk (stage T1bN2 monostation), IIIA, fit for surgery? | Which therapeutic approach would you recommend in patient with NSCLC in stage T1bN2 (no bulky pluristation), IIIA, fit for surgery? | | Which approach would you recommend in patient with NSCLC inoperable at diagnosis in partial response/stability (ycN2) after neoadjuvant cht? | Which approach would you recommend in patient candidate for chemo-radiation treatment with stage IIIA-B lung cancer? |
| --- | --- | --- | --- | --- | --- | --- |
| **Subgroups** | **N** | **Right answers % (P Value)** | | | |  |
| **Specialization:**  Radiation Oncology  Medical Oncology  Pneumology  Thoracic Surgery | 165  81  86  64 | 33 (p=0.001)  56 (p=0.008)  52 (p=0.04)  41 (p=0.726) | | 44 (p=0.00)  36 (p=0.360)  22 (p=0.033)  8 (p=0.00) | 58 (p=0.003)  49 (p=0.918)  41 (p=0.087)  36 (p=0.024) | 65 (p=0.00)  60 (p=0.181)  44 (p=0.042)  33 (p=0.000) |
| **Level of experience:**  0- 5 years  5-10 years  10-15 years  > 15 years | 117  48  78  156 | 32 (p=0.008)  44 (p=0.864)  41 (p=0.753)  51 (p=0.009) | | 29 (p=0.486)  35 (p=0.542)  31 (p=0.864)  33 (p=0.701) | 53 (p=0.289)  35 (p=0.047)  50 (p=0.824)  49 (p=0.876) | 47 (p=0.076)  60 (p=0.333)  50 (p=0.443)  59 (p=0.102) |
| **Dedicated working time:**  90-100%  70-90%  50-70%  <50% | 55  52  42  34 | 55 (p=0.059)  52 (p=0.041)  42 (p=0.970)  34 (p=0.03) | | 29 (p=0.629)  19 (p=0.005)  35 (p=0.390)  37 (p=0.063) | 39 (p=0.131)  43 (p=0.215)  54 (p=0.239)  52 (p=0.326) | 53 (p=0.902)  46 (p=0.123)  58 (p=0.267)  55 (p=0.715) |
| **Frequency of MTD:**  weekly  bi-weekly  not regularly  none | 43  50  37  36 | 43 (p=0.604)  50 (p=0.362)  37 (p=0.450)  36 (p=0.372) | | 32 (p=0.800)  15 (p=0.027)  42 (p=0.142)  33 (p=0.804) | 44 (p=0.004)  62 (p=0.116)  71 (p=0.004)  49 (p=0.984) | 56 (p=0.193)  41 (p=0.120)  42 (p=0.126)  62 (p=0.313) |
| **N of LA-NSLC pts in last year**  > 30  20-30  10-20  < 10 | 137  46  36  33 | 49 (p=0.066)  46 (p=0.453)  36 (p=0.082)  33 (p=0.182) | | 28 (p=0.334)  33 (p=0.652)  36 (p=0.267)  27 (p=0.452) | 45 (p=0.296  47 (p=0.598)  54 (p=0.163)  51 (p=0.750) | 50 (p=0.308)  52 (p=0.719)  56 (p=0.554)  62 (p=0.234) |
