# Supplementary material for: Management of locally advanced non-small cell lung cancer in the modern era: A national Italian survey on diagnosis, treatment and multidisciplinary approach
Source: PLoS One. 2019 Nov 13;14(11):e0224027. doi: 10.1371/journal.pone.0224027 (PMC6853329; doi:10.1371/journal.pone.0224027)
Supplement: Attachment — Submitted filename: Rebuttal Letter PLOSONE.docx [file pone.0224027.s007.docx]

Dear Editor,

thank you for the opportunity to revise and resubmit our manuscript entitled “Management of locally advanced non small cell lung cancer in the modern era: a national Italian survey on diagnosis, treatment and multidisciplinary approach”. According the reviewers advices, we revised our manuscript and highlighted all changes in the manuscript. The response to the reviewers are listed as follows. We hope that our modifications would meet your requirements.

We look forward to hearing from you, and are hopeful of a positive response.

Should you have any questions, please feel free to contact me.

Thank you and best regards.

Editor

I removed the tracked changes from page 6 of your manuscript

At page 13, I replaced “Table 2” and “Table 3” with “S3 Appendix” and “S4 Appendix” due to the complexity of the tables reported (as you suggested in a previous review).

I included captions for the supporting information files at the end of the manuscript, and I updated any in-text citations to match accordingly.

Reviewer #1

I submitted the paper to a native speaker to have a complete English language review and to correct all the grammatical and tense errors

To better explain what “fit” means, I maintained what we proposed as definition and I also reported the main features of patients enrolled in RTOG0617.

I added the overall response rate (in percentage of responders) of the survey at the beginning of graph “Results”

I reworded the Lines 168-170 that were confusing.

Together with all the co-aouthors, I tried to reduce the lenght of the graph “Discussion” underlying the most significative findings.

Reviewer #2

I modified the tense at page 6 line 123-128 trying to make it clearer

I accepted all the corrections/suggestions :

Page 12 line 229

Page 13 line 247

Page 13 line 250

Page 15 line 298

Reviewer #3

We reported in the text that answers were assumed as “correct” by the experts taking into account the main International Guidelines such as ESMO ones.
